# Processing of Larvae of *Alphitobius diaperinus* and *Tenebrio molitor* in Cooked Sausages: Effects on Physicochemical, Microbiological, and Sensory Parameters

**DOI:** 10.3390/insects15110843

**Published:** 2024-10-28

**Authors:** Barbara Lemke, Darleen Röpper, Anahita Arki, Christian Visscher, Madeleine Plötz, Carsten Krischek

**Affiliations:** 1 Institute of Food Quality and Food Safety, University of Veterinary Medicine Hannover, Foundation, Bischofsholer Damm 15, 30173 Hannover, Germanymadeleine.ploetz@tiho-hannover.de (M.P.); carsten.krischek@tiho-hannover.de (C.K.); 2 Institute of Animal Nutrition, University of Veterinary Medicine Hannover, Foundation, Bischofsholer Damm 15, 30173 Hannover, Germany

**Keywords:** processed raw insect larvae, *A. diaperinus*, *T. molitor*, cooked cured meat product, technological implementation, quality parameters

## Abstract

Proteins from insect production represent an interesting (environmentally friendly) option or supplement to commercial livestock farming. At present, however, the larval stages of *T. molitor* (mealworm) and *A. diaperinus* (buffalo worm) have been authorized as food for human consumption EU-wide, as have the nymph and adult stages of *Locusta* (*L.*) *migratoria* (*Locusta migratoria*, Linnaeus, 1758) and *Acheta* (*A.*) *domesticus* (house cricket, *Acheta domesticus*, Linnaeus, 1758). However, there is the problem that insects that are recognizable as a whole tend to be avoided by consumers, especially in the European region, as they are reminiscent of living things and can cause aversion and disgust in consumers. Against this background, in the present study, five batches of two types of cooked sausages were produced: on the one hand, with turkey, and on the other hand, with pork lean meat as a base. In different formulations, 10% and 20% of the meat contents (turkey or pork) in these meat products were replaced by deep-frozen, pulverized *T. molitor* and *A. diaperinus* larvae. The effects of the addition of these insects in the products on the microbiological and physicochemical parameters of these cooked sausages, compared to a product without insect content, directly after heating, were investigated. After production, a storage trial was also carried out to determine whether possible insect ingredients could influence the growth of inoculated bacterial species (*Bacillus* (*B.*) *cereus*, *Escherichia* (*E.*) *coli*, *Listeria* (*L.*) *monocytogenes*, *and Campylobacter* (*C.*) *jejuni*) and how the addition of insect larvae affectsthe sensory and physicochemical properties during storage. The study showed that the products with insects had reduced lightness (turkey *p* C = 0.025), increased yellowness (pork *p* S = 0.0009, *p* C < 0.0001 and turkey *p* C = 0.0027) and a reduced red color (pork *p* S < 0.0001, *p* C = 0.0001) after heating when compared to the cooked sausages without insects. However, no significant differences between the various cooked sausages with or without insects in terms of cooking loss, firmness, and protein, ash, and fat or water contents were found. The microbiological tests showed, on the one hand, that the prior microbial reduction (e.g., in the form of blanching) of the insect larvae was essential in order to guarantee the flawless microbiological quality of the cooked sausages and, on the other hand, that the addition of insects to the cooked sausages did not significantly affect the growth of the inoculated bacterial species and that no sensory differences could be detected during storage. Despite the significant color effects on the product, *A. diaperinus* and *T. molitor* larvae would be suitable as protein or meat alternatives in cooked sausages, but they would have to undergo pre-treatment, primarily with regard to microbiological safety. The extent to which a complete replacement of meat is possible has to be investigated in further studies.

## Simple Summary

Research on alternative protein sources is becoming increasingly important in our modern world due to the issue of sustainability and efficiency in food production. Insects are seen as promising alternatives to known animal protein sources due to their attractive nutritional composition and the fact that they can be bred and fattened in a possibly more resource-efficient way. As consumers, especially in Europe, have a certain disgusting feeling towards whole insects (larvae), various research projects are working on the production of hybrid (meat) products in which the insects are mostly processed as dried powders or flour so that they are unrecognizable to the consumer as a whole. In this study, in order to avoid the high-energy and complex steps of pre-processing, such as protein or fat extraction, drying, or hydrolysis, whole larvae of the species Alphitobius (A.) diaperinus (Alphitobius diaperinus, Panzer, 1797) and Tenebrio (T.) molitor (Tenebrio molitor, Linnaeus, 1758) were pulverized and then processed in a meat grinder (without pre-processing) together with the lean meat and other required ingredients. Five batches of two types of cooked sausages (turkey and pork cooked sausages) were produced: in addition to a control sausage without insect content, 10% and 20% of the turkey and pork lean meats were replaced with A. diaperinus and/or T. molitor larvae powder. The replacement with insect larvae had an influence on the color of the sausages, as well as on sensory properties such as smell and appearance. There was no effect on texture and nutritional values (e.g., fat and protein content). The processing of the insect larvae in the meat product also had no effect on the growth of inoculated bacterial species over a storage period.

## 1. Introduction

The constantly growing demand for animal protein due to the increase in the world’s population and (in some cases) growing prosperity (e.g., in China, India) can no longer be adequately met by current food production conditions in the future [1]. The amount of meat produced annually is expected to rise from approximately 350 million to 410 million tons by 2050 [1] as it is expected that the world’s population will increase to 9.7 billion people by 2050, in addition to the growing prosperity [2]. It is therefore necessary to find food alternatives or other sources of nutritional protein considering that these protein sources are easy to find and produced sustainably. Both meat products as well as bakery products are often studied with regard to the addition of insects as meat protein alternative [3]. Insects are seen as promising options for covering the increasing protein demand of the world’s growing population. Some insect larvae, such as the *T. molitor* larvae, primarily develop on dry grains and cereals, but can be reared on organic substances, such as fruit and vegetable scraps, as well as on substances that arise during the beer brewing process or by-products of the bread baking process [4]. As, in many countries, food, especially comprising fruits and vegetables, is often wasted, being discarded and rejected when it does not meet the standard or has just reached its expiry date, and as this food is usually of sufficient to excellent quality, the use of these side-stream components might be an interesting feeding alternative for the production of insects worldwide. However, as, at present in the European Union, insect larvae must not be fed with catering waste or food waste containing meat and fish [5], the rearing with ‘waste’ containing fruit and vegetables is also possible in the European Union with the disadvantage that the food waste has to be carefully separated.

Insect breeding and rearing is a sustainable alternative to commercial livestock farming [6,7]. The high nutrient density of insects also makes them also interesting alternatives to conventional protein and fat sources. *Alphitobius* (*A.*) *diaperinus* and *Tenebrio* (*T.*) *molitor*, for example, have protein contents of approx. 50–60% [3,8], and *T. molitor* contains all essential amino acids [9]. The lipid content of *A. diaperinus* is about 29%, and that of *T. molitor* is between about 17% and 25% [8,10]. For example, the fatty acid compositions of insects can be compared with those of fish regarding their degree of unsaturation [11]. Insects are rich in long-chain, unsaturated fatty acids [10] and *A. diaperinus* and *T. molitor* show contents of saturated fatty acid of approx. 41% and 30%, of mono-unsaturated fatty acids of approx. 38% and 39%, and of poly-unsaturated fatty acids of 22% and 32% [8]. In addition, *T. molitor* could be a promising source of zinc and magnesium [12], as well as of copper and calcium [9]. Additionally, insects (larvae) in general are sources of riboflavin and biotin and *T. molitor* contains about 16% fiber, which could be a good supplement in the human diet to strengthen the gut microbiome [3]. However, the nutritional value of insect species depends on factors like the species, feeding, developmental stage, or rearing process [8,13,14,15]. Beetle larvae, like those of *T. molitor*, *A. diaperinus*, and *Zophobas* (*Z.*) *morio*, make up the majority (31%) of the total number of edible insects [16]. Replacing meat protein with other protein sources is important as obtaining protein from insects in food products is an environmentally friendly way of reducing commercial livestock farming with its associated problems [6].

Insects can be offered as whole animal carcasses (larva, imago), but the consumer acceptance for these in Europe, in contrast to other continents such as Asia, is lower. Insects that are recognizable as a whole are more likely to be avoided by European consumers [17]. According to Martins et al. [18], whole insect larvae are suggestive of life and cause increased aversion and disgust among consumers. In order to overcome this problem, insects can be pulverized as finely as possible and directly used in products, known to the consumer, like sausages or, for example, used after fat and/or protein extraction in products. For example, investigations have been made with *T. molitor* as a source of enrichment in animal-based [19] and plant-based [19,20] burger patties, and *T. molitor* has also been examined for use in wheat bread [21] and in maize-based tortillas [22]. However, not only must attention be paid to the insect with regard to disgust, but possible consumer’s concerns with regard to chemical and microbiological contamination have also to be considered. The risk of the chemical and microbiological contamination of the insects depends, for example, on the one hand, on the feeding, and on the other hand, on environmental conditions. Various microorganisms, such as *Pseudomonas aeruginosa* and *Staphylococcus aureus*, representatives of the pathogenic *Bacillus cereus sensu lato* group, as well as apathogenic *Bacillus* spp. [23,24], have been isolated from the cuticle of insects (larvae). Furthermore, *Salmonella* spp. and *Shigella* spp. [25], as well as *Campylobacter* spp. [25,26] and *Listeria monocytogenes* [24,26], have been detected in and on insects. In studies, human pathogenic microorganisms such as *Campylobacter* spp., *Salmonella* spp., and *Shigella* spp. were detected more frequently if insects were taken from unclean environments [25]. Concerns about allergies must be also taken into account; these can occur due to the chitin shell or the tropomyosin protein complex also known from allergies caused by crustaceans [3].

In many previous studies investigating ‘hybrid meat products’, such as cooked sausages with *T. molitor* larvae, the insect larvae were subjected to prior technological treatments like freeze-drying [27], spray-drying [28], or protein extraction [29]. The main objective of this study was to investigate the effect of replacing pork and turkey meat in cooked sausages (‘mortadella-style’) with powders from whole, untreated, deep-frozen insect larvae of *T. molitor* and *A. diaperinus* on the sensory, physicochemical, and microbiological parameters of these meat products compared to a conventional product without insect addition. However, we did not want to produce a comparable product. An additional aim was to avoid energy-intensive and complex pre-processing steps with the insect larvae because we wanted to keep the processing as simple as possible, considering an applied approach. Additionally, these analyses were performed not only after the preparation of the sausages but also during a storage trial in which sausage slices were packaged in a modified atmosphere (MAP), inoculated with bacteria before packaging, and stored for up to 14 days. In addition to the influence of the insect species, the impacts of different concentrations of the insects in the hybrid (meat) product (10%, 20%) were also taken into account.

## 2. Materials and Methods

### 2.1. Experimental Design: Technological Realization in Manufacturing, Packaging, and Storage

Frozen insect larvae of the species *A. diaperinus* (buffalo worm) and *T. molitor* (mealworm) were purchased from an insect company (Fauna Topics Zoobedarf, breeding and trade LLC, Marbach am Neckar, Germany). *A. diaperinus* and *T. molitor* larvae used in our experiments were subjected to European feed law and therefore did not have European food law authorization. They may be fed to domestic and fur animals and to certain livestock species, like poultry, pigs, and fish from aquaculture (regulation (EG) 1069/2009, regulation (EU) 142/2011). At the end of the rearing period, the larvae were killed at the company Fauna Topics Zoobedarf by freezing at −25 °C, transferred to plastic bags, and sent overnight in Styrofoam boxes with dry ice to the Institute of Food Quality and Food Safety. In the institute, the larvae were stored in a freezer at −20 °C until use. After delivery to the institute, the insect larvae were stored at −20 °C. In preparation for the sausage production, the larvae were pulverized for 1 min (minute) at 10,000 round per min (rpm) (Grindomix GM 200, Retsch GmbH, Haan, Germany), packaged in plastic bags, and then again stored at −20 °C until use. Pulverized samples for analysis of the nutrient and fatty acid composition were individually packaged in plastic bags and stored at −20 °C.

For the production of the meat products in three independent repeats, “mortadella-style” cooked sausages with five different formulations, with either pork or turkey meat, were produced, considering the recipes shown in Table 1. Different formulations of meat products were made, replacing 10% or 20% of the pork (p) and turkey meat (t) with pulverized larvae of *A. diaperinus* (t-A 10/20, p-A10/20) and *T. molitor* (t-T 10/20, p-T 10/20). The turkey meat control (t-control) and pork (p-control) were produced without any insects. The percentages of 10% and 20% were chosen as preliminary tests at the institute had indicated that replacing more lean meat with insect powders resulted in quality alterations of the products, mainly with regard to softening, probably due to inadequate protein coagulation. Pork shoulders and turkey thighs were chosen as meat sources for the cooked sausages. Pork back fat was chosen as the fat source for all sausages. Meat and fat were freshly purchased from a German commercial slaughterhouse, approx. one week before production, to minimize potential negative effects of freezing. Both meat and fat were separately homogenized and then stored at –20 °C in a plastic bag until use. Meat and fat were removed from the freezer and thawed 24 h before production. For the production of the cooked sausages, at first the meat, the insect powders (if applied), the other ingredients (except fat, Table 1), and half of ice were homogenized (‘other ingredients’ and ice were purchased from Hannoversche Gewürzmühle, Hanover, Germany). After that, the fat and the residual ice were added and the mixture was further homogenized. The mixture was then transferred to artificial casing with a caliber of 50 mm and a length of 400 mm (NALO sausage casing, Hannoversche Gewürzmühle, Hanover, Germany), weighed, and cooked at +75 °C for 70 min to ensure a meat-product core temperature of +72 °C. After that, the sausages were cooled and weighed again to calculate the cooking loss.

**Figure 1:**
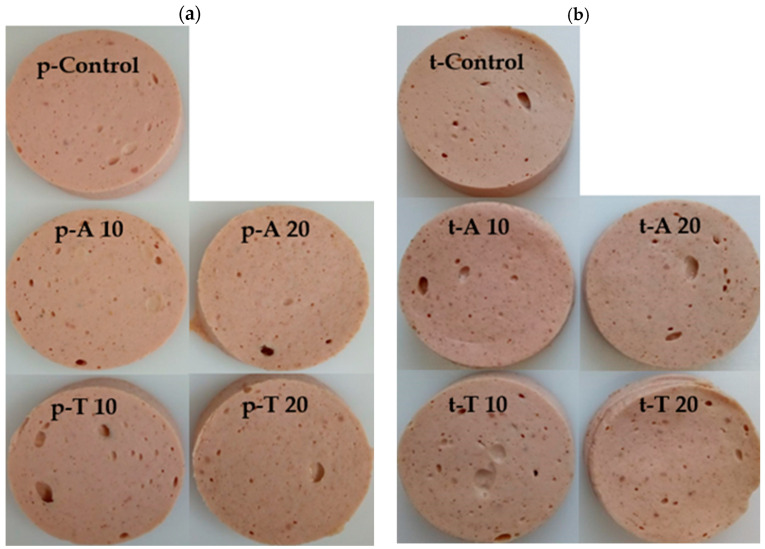
Images of the pork cooked sausages (**a**) and turkey cooked sausages (**b**) after cooling and slicing. p/t-: pork or turkey cooked sausages; control: without insect larvae powder (top); center, left to right—p/t-A 10/-A 20 consisted of 10%/20% pulverized *Alphitobius diaperinus* larvae; bottom, left to right—p/t-T 10/-T 20 consisted of 10%/20% pulverized *Tenebrio molitor* larvae.

For the storage experiments, the cooked sausages, used for analysis of the physicochemical parameters, were cut in slices (50 mm in diameter and 3.5 mm thick, Figure 1). The slices were packaged in modified atmosphere (MAP, MULTIVAC, Typ T 100, Wolfertschwenden, Germany). Therefore, the samples were transferred to polypropylene trays (ES-Plastic GmbH, Hutthurm, Germany) and sealed with a polyethylene-ethylene vinyl alcohol PP transparent film (Südpack, Ochsenhausen, Germany) after vacuumizing and refilling with the appropriate gas composition (70% N_2_, 30% CO_2_).

On storage days 0, 7, and 14, a third of the packages were randomly chosen and the plastic film was opened. Directly after opening, the sensory properties and the color and pH values were determined. After homogenizing the samples (Grindomix, Retsch, Haan, Germany) at 6.500 rounds for 7 s, the water activity (a_w_) and nutritional composition (only day 0) using a NIR were determined. Homogenized samples for the analysis of the antioxidative capacities (only day 0 and 14) were stored in plastic bags at −20 °C until analysis.

### 2.2. Preparation of the Inocula (Microorganisms) and Inoculation of the Sausage Slices

The microorganisms for the inoculation experiments, *B. cereus* (DSM 4222), *E. coli* (DSM 682), *L. monocytogenes* (DSM 20600), and/or *C. jejuni* (DSM 4688), were all procured from the German Collection of Microorganisms and Cell Cultures (DSMZ, Braunschweig, Germany) and stored in cryotubes at −80 °C. Three days before the inoculation experiments, the frozen bacteria isolates were transferred to sheep blood agar (Oxoid GmbH, Wesel, Germany) and incubated at +30 °C for 48 h, except for *C. jejuni. C. jejuni* was incubated at +42 °C in microaerophilic atmosphere 20 to 24 h before the experiment. Individual colonies from *B. cereus*, *E. coli*, and *L. monocytogenes* were transferred into brain–heart–glucose–bouillon (BHI, Carl Roth GmbH & Co. KG, Karlsruhe, Germany) and incubated at 30 °C. Colonies of *C. jejuni* were transferred to sheep blood agar and incubated for 24 h at +42 °C in microaerophilic atmosphere. On the inoculation experiment days, colonies of *C. jejuni* were transferred to sterile saline solution with peptone (NaCl-P, 0.85% NaCl and 0.1% peptone) and the absorption was measured with a densitometer (Densimat, BioMérieux, Marcy-l’Etoile, France). A solution with a McFarland turbidity unit of 3.0 was used for the *C. jejuni* inoculation. The BHI broths with the other bacteria after incubation for 20 to 24 h were utilized for the other inoculation experiments.

The total number of bacteria in the McFarland solution and the BHI broth was analyzed on every examination date. Therefore, the solution was diluted in NaCl-P up to 10^7^ and 0.1 mL of the appropriate dilution was spread on the specific selective agar plates as described below (Section 2.3.5).

For the inoculation experiments, the surface of each of the sausage slices (50 mm in diameter and 2.5 mm thick, weight of approx. 10 g) was inoculated with 100 µL of *Bacillus* (*B.*) *cereus*, *Escherichia* (*E.*) *coli*, *Listeria* (*L.*) *monocytogenes* (only pork sausages), and *Campylobacter* (*C.*) *jejuni* (only turkey meat sausages). The inoculum was distributed on the surface with an L-shaped spreader (VWR International, Darmstadt, Germany). The sausage slices were inoculated with approx. 10^5^ colony forming units (cfu) and then packaged in MAP as described above. The packages were stored in a freezer at +6 °C for up to 14 days.

### 2.3. Methods

#### 2.3.1. Nutrient Analysis

After production (day 0), the fat and protein contents of the unprocessed insect larvae, as well as their contents of ash and moisture, were determined as follows.

To determine the fat content, a Soxhlet apparatus (LAT GmbH, Garbsen, Germany), following the norm ISO 1443:1973 (ISO, International Organization for Standardization, Geneva, Switzerland), was applied. The acid hydrolysis and extraction was accomplished in triplicates and the mean value was used for statistical analysis.

To calculate the protein concentration, we detected the nitrogen content, applying the Kjeldahl method (ISO 937:1978), using a Vapodest 50s (Gerhardt Laboratory Systems GmbH, Koenigswinter, Germany). For the calculation of the protein content, the nitrogen content was multiplied with 6.43 (*A. diaperinus*) and 5.2 (*T. molitor*) [30].

The content of ash was determined by taking 4 to 5 g (weight accuracy: ±1 mg) of the homogenized sample and combusting the material at +550 °C for 5 h. After cooling the combusted material for 1.5 h in a desiccator and reweighing ±1 mg), the ash content was calculated with the following formula:

$$\mathrm{Ash}\;\left(\%\right)=\frac{\left(m2-m1\right)\times 100}{m0}$$

Here, *m*_0_ designates the initial weight of the sample, *m*_1_ the mass of the dried vessel, and *m*_2_ the mass of the vessel with the ash after heating (L 06.00-4:2017-10, procedure specified by the German Federal Office of Consumer Protection and Food Safety, based on §64 German Food and Feed Law).

The moisture content and the dry matter (100 − moisture) were determined as follows. A vessel, filled with 30 g sand and a glass rod, was weighed and 4 to 5 g (accuracy: ±1 mg) of the homogenized sample was added. After intensive mixing of the sand and the material with the glass rod, the mixture was dried for 4 h at +103 °C. After that, the samples were cooled in a desiccator and reweighed (accuracy: ±1 mg). The moisture content was calculated with the following formula:

$$\mathrm{Moisture}\;\left(\%\right)=\frac{\left(m0-\left(m2-m1\right)\right)\times 100}{m0}$$

Here, *m*_0_ describes the weigh-in of the sample; *m*_1_ the empty weight of the bowl, the glass rod, and the sand; and *m*_2_ the mass of the bowl, including sand, glass rod, and dried sample (L 06.00-3:2014-08, procedure specified by the German Federal Office of Consumer Protection and Food Safety, based on §64 German Food and Feed Law).

Using near-infrared apparatus (NIR, model TANGO, Bruker Corporation, Leipzig, Germany) the contents of the nutrients, ash, protein, fat, and sodium chloride and the moisture percentages of the homogenized cooked sausages were determined on day 0.

#### 2.3.2. Fatty Acid Analysis

The determination of the fatty acids was performed by the Institute for Animal Nutrition according to a modified method shown in the publication by Lepage and Roy in 1986 [31]. Samples of the homogenized insect larvae and the homogenized cooked sausages (after production) were analyzed. Therefore, 200 to 300 mg of the samples were weighed in glass tubes and blended in a ratio of 1:20, depending on the weigh-in, with a mixture of methanol-benzene (4:1), containing tridecanoic acid (C 13:0) as an internal standard. Since fatty acid methyl esters (FAMEs) are determined in this method, 400 µL to 600 µL (depending on the weigh-in) acetyl chloride was added for transesterification, followed by a one-hour cooking step and centrifugation period of 15 min. The supernatant was transferred to tubes to analyze the fatty acid composition using a gas chromatograph (Type Trace 1300, Thermo Fisher Scientific, Dreieich, Germany) containing a flame ionization detector. The liquid sample evaporated at 280 °C and was passed through the capillary column (Type RT-2560, Restek GmbH, Bad Homburg, Germany) using nitrogen as a carrier gas. After the temperature in the column was kept at 50 °C for 2 min, the temperature was gradually increased to 240 °C with the following temperature program: starting temperature of 50 °C for 2 min; temperature increase to 120 °C at a rate of 30 °C per min; increase to 240 °C at a rate of 3.5 °C per min. The final temperature of 240 °C was maintained for 12 min. The analysis was carried out in duplicates. The detection limit was 0.05 g/kg. Both the total fatty acid (TFA) contents of the original (“fresh”) substance of the insects or the meat products as well as the saturated fatty acids (SFAs), the mono-unsaturated fatty acids (MUFAs), and poly-unsaturated fatty acids (PUFAs), with a chain length from C 4:0 up to C 22:0 (C 22:6), were detected. The TFA was determined from the sum of all detected fatty acids and was therefore, strictly speaking, not the total fatty acid content but kind of approximate value. SFA, MUFA, and PUFA were evaluated in % of TFA.

#### 2.3.3. Physicochemical Analysis

After production, the percentual cooking loss (in %) of the sausages was calculated by subtracting the weight after cooking from the weight before cooking, followed by dividing this result to the weight before cooking and multiplication with 100.

To determine/calculate the hardness (in Newton, N), the cohesion (in %), and the elasticity (in %) of the cooked sausages after cooking, the texture analyzer Ta.XT.plus (Stable Micro Systems, Survey, Godalming, UK) was used. The analyzer consisted of a 50 kg power cell and a round aluminum stamp (50 mm), which moved down during every experiment with a speed of 3 mm/s until 40% of the sample height was reached and finally up again with a speed of 3 mm/s. For the measurements, each of the sausage slices had a thickness of 25 mm and a diameter of 22 mm. The measurements were performed in triplicates, and again, the mean values were used for statistical analysis.

Using a colorimeter (Konica-Minolta GmbH, Langenhagen, Germany, 8 mm measuring field, standard observer, D65 illuminant), the color values of lightness (L*), redness (a*), and yellowness (b*) of the cooked sausages after cooking and on storage days 0, 7, and 14 were determined. Before measurement, the apparatus was calibrated with a white plate (Konica-Minolta GmbH). The color was measured three times and for statistical analysis, the mean value was used.

The water activity (a_w_) of the cooked sausages were determined with an a_w_-cryometer (AWK-40, NAGY Messsysteme GmbH, Gäufelden, Germany) on storage days 0, 7, and 14. To check the correct functionality of the cryometer, a saline solution with 5% concentration was used. The measurements were performed once.

To determine the pH values of the sausages during storage, a Portamess pH meter (Portamess^®^, Knick GmbH, Berlin, Germany) with a temperature sensor and a glass electrode (InLab 427, Mettler-Toledo, Urdorf, Switzerland) was used. For calibration, we utilized standard solutions with pH 4.0 (±0.02) and pH 7.0 (±0.01) from the company Carl Roth GmbH & Co. KG (Karlsruhe, Germany). The pH value was determined in duplicates and for statistical analysis, the mean value was used.

The antioxidant capacity of the cooked sausages was detected during storage on days 0 and 14 using a combination of different methods. For the production of the radical solutions, we applied the method of Re et al. [32]. Therefore, a 7 mM 2.2′-Azino-di-[3-ethylbenzthiazoline-6-sulfonic acid (ABTS) solution (Fischer Scientific GmbH, Schwerte, Germany) and a 26.95 mM potassium persulfate solution (VWR International GmbH, Darmstadt, Germany) were diluted 1:11 and incubated in the dark at room temperature for 12 to 16 h. On the examination day, the radicalized solution was diluted with distilled water to optical density 0.7 ± 0.02 using a photometer (Evolution 201, UV-Visible Spectrophotometer, Fischer Scientific GmbH) and an absorption wavelength of 734 nanometer. The homogenized, frozen sausage samples were thawed, weighed to approx. 1.0 g, homogenized with 6 mL distilled water for 1 min on ice, then shaken on ice for one hour and centrifuged for 15 min at 2.340× *g*. The supernatant was removed and 20 µL of the supernatant was pipetted into a cuvette and mixed with 3 mL of the radicalized ABTS solution. This solution was incubated for 7 min at room temperature in the dark and then measured at wavelength of 734 nm. Solutions for the calibration curve were prepared, according to Re at al. [32], using Trolox^®^ [6-Hydroxy-2.5.7.8-tetramethylchroman-2-carbonsäure, 97%] (Thermo Fisher Scientific, Dreieich, Germany) in concentration between 0 und 15 µM and the solutions were analyzed in the same way as the supernatants described in the previous sentence. The antioxidant capacity was given in terms of µM (micromole) Trolox Equivalent antioxidant capacity (µM TEAC/g sausage), considering the calibration curve and the following formula:

$$\mathrm{Antioxidant}\;\mathrm{capacity}\;\left(µM\;\mathrm{TEAC}\;/\;g\;\mathrm{sausage}\right)=\frac{\mathrm{TEAC}\;\mathrm{concentration}\;\left(µM\right)}{\mathrm{sample}\;\mathrm{weight}\;\left(\mathrm{gram}\right)\;}$$

#### 2.3.4. Sensory Analysis

On storage days 0, 7, and 14, the non-inoculated packages were observed from the outside and the sausage slices were rated from a scale from 1 to 5 in relation to their appearance (modified test from the German Agricultural Society, DLG). No deviations were rated with 5 points, minor deviations with 4 points (expert recognizes differences), moderate deviations with 3 points (average consumer recognizes differences with high probability), significant deviations with 2 points (layperson recognizes deviations in the product), and severe deviations with 1 point (not fit for consumption). After that, the packages were opened and appearance and odor were immediately evaluated, again referring to the described 1-to-5 scaling. The sensory analysis was carried out by three trained persons. As the appearance has a higher impact on the consumer’s buying decision, the appearance was more considered. The total sensory analysis was calculated by multiplying the appearance average of the three persons with 3 and the odor average with 1. This result was divided by 4 to achieve a final sensory score value between 1 and 5.

#### 2.3.5. Microbiological Analysis

The native, unprocessed insect larvae (*A. diaperinus* and *T. molitor* larvae) were microbiologically analyzed regarding the total count of viable aerobic microorganisms (TVC) and number of yeasts and mold fungi. Therefore, 10 g of the thawed, whole, unprocessed larvae was weighed in the Stomacher plastic bags and treated as described before. To detect the yeast and mold fungus numbers, dilution series up to 10^5^ were prepared and 0.1 mL of the dilutions was pipetted onto YGC (Yeast Extract Glucose Chloramphenicol) agars (Oxoid GmbH, Wesel, Germany), which were used as selective culture media to detect yeast and mold fungus. The plates were incubated at +25 °C for five days (ISO 11133:2014).

On storage days 0, 7, and 14, 10 g of the inoculated sausage samples was transferred to plastic bags (Stomacher 400 Strainer Bag, Seward, AK, USA), filled with a NaCl-P in relation 1:10, and homogenized at 230 rounds per min for 2 min with a stomacher (Stomacher 400 Circulator, Seward Limited, Worthing, UK). After that, serial dilutions up to 10^6^ were prepared and 0.1 mL of appropriate dilutions was transferred to the specific selective culture medium and carefully spread with a spatula. The selective culture media (Oxoid GmbH, Wesel, Germany) were as follows: PEMBA (Polymyxin–Egg-Yolk–Mannitol–Bromothymol Blue) agar, *B. cereus* selective agar, ISO 7932:2004), Chromogenic Coliform agar (ColiC agar, *E. coli* selective agar; ISO 16649-2:2001), Oxoid Chromogenic Listeria agar (OCLA, *L. monocytogenes*, ISO 11290-1:2017), and CCD (Charcoal Cefoperozone Deoxycholate) agar (*C. jejuni* selective culture medium without blood, ISO 10272-1, 2:2017). All microbial plates, except CCDA, were incubated at +30 °C for 48 h. CCDA plates were incubated in microaerophilic atmosphere at +42 °C for 44 h.

Additionally, the total viable number (TVC) of the non-inoculated sausage slices was determined on storage days 0, 7, and 14 using plate-count agar (Carl Roth^®^ GmbH & Co. KG, Karlsruhe, Germany, ISO 4833:2003). After dissolving 23.5 g of the plate-count agar powder in 1 L distilled water, the agar was treated in a steaming pot at approx. +110 °C for one hour and then slowly cooled down in a water bath to +50 °C. Ten gram of the sausage slices were weighed, filled up with NaCl-P to the ninefold, and homogenized as described before. A quantity of 1 mL of this powder was pipetted into a sterile petri dish and approx. 15 mL of the PC agar was added. After that, the dishes stood until the agar was solid and were then incubated at +30 °C for 72 h (ISO 4833:2003).

The detection limits for the TVC were 0.7 log_10_ cfu/g, and for the other microorganisms, 1.7 log_10_ cfu/g, which was used for further calculations if no colonies were found on the agars.

### 2.4. Statistical Analysis

For statistical analysis, SAS Enterprise Guide 7.1 (SAS Institute Inc., Cary, NC, USA) was used. The data were analyzed with the mixed model, considering the following models:

Native unprocessed insects:

Y_ij_ = µ + S_i_ + R_j_ + ε_ij_

Cooked sausages:

Y_ijk_ = µ + S_i_ + C_j_ + SC_ij_ + R_k_ + ε_ijk_

Y_ij_ = µ + MS_i_ + Rj + ε_ij_

Y_ijk_ = observation value; µ = overall mean; S_i_ = fixed effect of the insect species (*A. diaperinus*, *T. molitor*) or control (only sausage experiments); C_j_ = fixed effect of insect concentration (0, 10, 20%); SC_ij_ = fixed effect of interaction of S and C; MS_i_ = fixed effect of meat source (pork or turkey lean meat) and insect species; R_j/k_ = random effect of repeat; ε_ij/ijk_ = random error.

If the F test in Section 3.2 and Section 3.3 was significant (*p* ≤ 0.05), the significant differences between the individual means were calculated with the Tukey multiple-means comparison test. All values were presented as means ± standard deviations (SDs). Differences between the groups were significant if the *p*-value with the Tukey test was lower than 0.05.

## 3. Results

### 3.1. Analysis of the Unprocessed Insect Larvae

#### 3.1.1. Nutrient Analysis

The ash, protein, and total water (or dry matter) results of the two insect species did not differ significantly (*p* > 0.05). *A. diaperinus* larvae showed significantly (*p* ≤ 0.05) lower fat content than *T. molitor* larvae (Table 2).

#### 3.1.2. Fatty Acid Analysis

Analyzing the fatty acid composition of the insect larvae (Table 3), TFA and PUFA contents were significantly (*p* ≤ 0.05) higher and those of SFAs significantly (*p* ≤ 0.05) lower in *T. molitor* larvae compared to the *A. diaperinus* larvae. *T. molitor* larvae had significantly (*p* ≤ 0.05) higher percentages of linoleic acid (C 18:2, cis-9,12), palmitoleic acid (C 16:1), palmitic acid (C 16:0), myristic acid (C 14:0), and lauric acid (C 12:0) and significantly (*p* ≤ 0.05) lower stearic acid (C18:0) percentages than the larvae of *A. diaperinus*.

#### 3.1.3. Microbiological Analysis

During microbiological examination, the TVC results and numbers of yeasts or fungi did not differ significantly (*p* > 0.05) between the insect larvae (Table 2).

### 3.2. Analysis of the Cooked Sausages After Production and During Storage in Modified Atmosphere Packages

#### 3.2.1. Nutrient Analysis of the Cooked Sausages After Production

**Figure 2:**
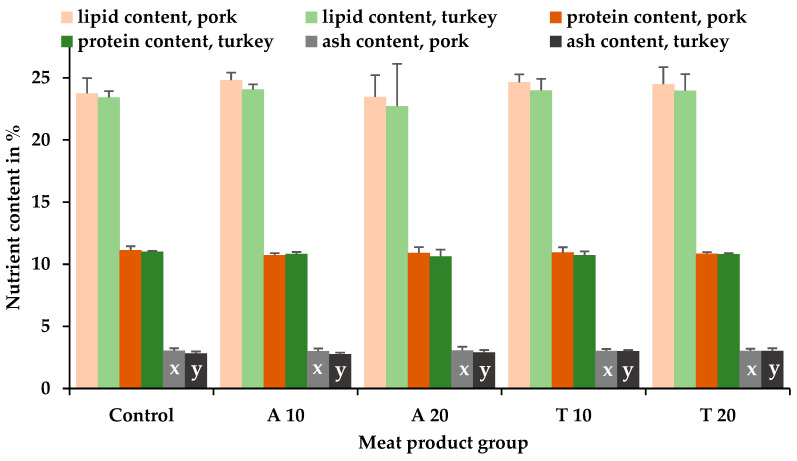
Mean and positive standard deviation values of the fat, protein, and ash contents of the different cooked sausages (in % of the fresh/original substance) on storage day 0 depending on the meat product group and the meat source (pork versus turkey meat). Meat product groups: control (without insect powder); A 10/A 20, consisting of 10%/20% pulverized *A. diaperinus* larvae; T 10/T 20, consisting of 10%/20% pulverized *T. molitor* larvae. ^xy^ bars with different letters between the animal meat sources (N = 6) (pork and turkey meat) within the same insect species were significant (*p* ≤ 0.05).

After production (day 0), the protein, fat, and ash contents in the pork- and turkey-meat cooked sausages did not differ significantly (*p* > 0.05) between the insect species and the controls, the concentrations of the insects, and the interaction of insect species and concentrations (Figure 2).

#### 3.2.2. Fatty Acid Analysis of the Cooked Sausages After Production

The percentages of some fatty acids, detected on day 0, in cooked sausages are shown in Table 4. The turkey-meat cooked sausages showed no significant differences in the fatty acids with regard to the insect species, insect concentrations, and their interaction (*p* S, C and S*C > 0.05) whereas the pork sausages were not significantly influenced by the insect concentration or the interaction of species and concentration (*p* C and S*C > 0.05). However, pork cooked sausages containing *T. molitor* larvae powder had significantly (*p* S ≤ 0.05) higher lauric acid results than the products containing *A. diaperinus* or the control products.

#### 3.2.3. Physicochemical Analysis

##### Cooking Loss of the Cooked Sausages After Production

The cooking losses of the cooked sausages after production (day 0), produced with pork and turkey, showed no significant differences (*p* > 0.05) considering the insect species, the different concentrations, or their interaction (Table 5). Products containing 20% insect larvae tend to have a higher cooking loss (*p* C = 0.0529) than turkey products, containing 10% insect larvae and the turkey control products.

##### Textural Profile Analysis of the Cooked Sausages After Production

**Figure 3:**
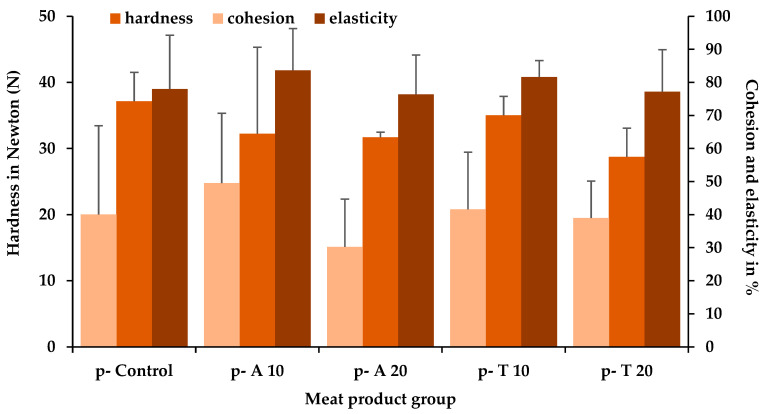
Mean and positive standard deviation values of the texture profile analysis of the pork cooked sausages after cooking depending on the meat product group: p-control (without insect powder); p-A 10/-A 20, consisting of 10%/20% pulverized *A. diaperinus* larvae; p-T 10/-T 20, consisting of 10%/20% pulverized *T. molitor* larvae.

With regard to the texture, no significant (*p* > 0.05) differences in hardness, cohesion, and elasticity between the pork products with and without insects could be found, considering the insect species, the concentrations, and their interaction (Figure 3). Similar results were found with the turkey meat products, which also showed comparable hardness (37.2 ± 3.2 N), cohesion (61.2 ± 5.6%), and elasticity (86.7 ± 1.8%) results.

##### Color Analysis

- Cooked sausages after production

When examining the Lab* values, considering the interaction between each insect species and its concentration, no significant differences (*p* S*C > 0.05) were found between the groups of cooked sausages made with pork and turkey meat (day 0, Table 6).

L* values revealed no significant differences (*p* S > 0.05) between the pork and turkey meat sausage groups, considering the insect species and the insect concentration (only pork). Turkey meat products with a 20% insect content had significantly (*p* C ≤ 0.05) lower L* values than products with 10% insects and t-control (day 0, Table 6).

Whereas a* values of the turkey meat products were not significantly influenced by each insect species and its concentration, pork control sausages showed significantly (*p* S ≤ 0.05) higher a* values compared to the pork insect products. Considering the concentration, pork sausages without an insect content (0%) had significantly (*p* C ≤ 0.05) higher a* values than the products with 10% and 20% insect powder (*p* C > 0.05) (day 0, Table 6).

Furthermore, pork products had significantly (*p* S ≤ 0.05) higher b* values compared to the p-control, independent of the insect species. Considering the effect of the insect concentration, pork and turkey sausages containing 20% insects had significantly (*p* C ≤ 0.05) higher b* values than sausages with 10% insect larvae and *p*-control. Interestingly, pork control sausages had significantly lower (*p* C ≤ 0.05) b* values compared to the products containing 10% insect larvae of *T. molitor* and *A. diaperinus* (day 0, Table 6).

- Cooked sausages during storage in modified atmosphere packages.

Regarding the interaction of species and concentration, there were no significant differences (*p* S*C > 0.05) in the color (Lab*) for the cooked turkey meat and pork sausages on storage days 7 and 14 (Table 6).

Considering the statistical impact of the insect species and their concentration, pork and turkey meat products showed comparable L* results on days 7 and 14 of storage (Table 6).

With regard to the impact of the insect species on the a* values, on storage day 7, all pork products differed significantly (*p* S ≤ 0.05) from each other, with p-control having the highest a* value, followed by the *T. molitor* products and the sausages containing *A. diaperinus*. In contrast, regarding the turkey products on storage day 7, control products had only significantly higher (*p* S ≤ 0.05) a* values than turkey products containing *A. diaperinus* (*p* S ≤ 0.05). The a* results of the sausages with *T. molitor* did not differ significantly (*p* S > 0.05) from those of the other groups. On storage day 14, pork and turkey meat control sausages had significantly higher (*p* S ≤ 0.05) a* values than the corresponding insect products independent of the insect species (Table 6). Furthermore, on storage days 7 and 14, considering the factor concentration, *p*-control showed significantly higher (*p* C ≤ 0.05) a* values (*p* C ≤ 0.05) than 10% and 20% pork insect products, whereby the a* results of the groups containing 10% and 20% powder did not differ significantly (*p* C > 0.05) from each other. In contrast, on storage day 7, t-control had significantly higher (*p* C ≤ 0.05) a* values than 20% turkey–insect products, but products containing 10% insects differed not significantly (*p* C > 0.05 from t-control and the 20% insect sausages. Like in the pork products on storage day 14, a* values in t-control were significantly higher (*p* C ≤ 0.05) compared to the products with 10 and 20% insect larvae (Table 6).

With regard to the factor insect species, pork products with insect content showed significantly higher (*p* S ≤ 0.05) b* values during storage days 7 and 14 than the control products. This effect was not found on day 7 in the turkey meat products. On storage day 14, insect products, independent of the species, again had significantly higher (*p* ≤ 0.05) b* values than the control sausages. Considering the factor concentration, on all storage days, 0%, 10%, and 20% pork products differed significantly (*p* C ≤ 0.05) from each other. The 20% insect products had the significantly (*p* C ≤ 0.05) highest b* value, followed by the 10% and the 0% pork products. In contrast, the b* values of the turkey meat groups were comparable (*p* C > 0.05) on storage day 7 whereas on day 14, the effect of the concentration was similar to what was found in the pork products, with the significantly highest (*p* C ≤ 0.05) b* values in the 20% turkey meat sausages, followed by the 10% and 0% turkey meat sausages (Table 6).

##### Water Activitiy (a_w_-Value), pH Value and Antioxidant Capacity

- Cooked sausages after production

There were no significant differences (*p* > 0.05) between the a_w_-values of the pork products (0.9732 ± 0.001) after production, considering each insect species, its concentration, and their interaction.

In contrast, the turkey meat products containing *A. diaperinus* had significantly higher (*p* S ≤ 0.05) a_w_-results after cooking than the *T. molitor* and the control sausages (0.9714 ± 0.001).

Regarding the insect species, its concentration in the product, or their interaction after production, the pH values and the antioxidant capacities were comparable (*p* > 0.05) between the different pork- and turkey-meat cooked sausage groups. Pork products showed mean pH values of 5.9 ± 0.01 and turkey meat products of 6.1 ± 0.1. The antioxidant capacities were 4.8 ± 0.7 µM TEAC/g in pork sausages and 2.9 ± 0.5 µM TEAC/g in turkey meat products.

- Cooked sausages during storage in modified atmosphere packages

On storage days 7 and 14, no significant differences regarding the a_w_-value results in the pork and turkey meat sausages considering the species (*p* S > 0.05), the concentration (*p* C > 0.05), and their interaction (*p* S*C > 0.05) were found. Pork-based products had a_w_-values of 0.9727 ± 0.001 on day 7 and 0.9698 ± 0.01 on day 14. Turkey meat products had a_w_-values of 0.9715 ± 0.001 on day 7 and 0.9719 ± 0.001 on day 14.

Regarding the insect species, its concentration in the product, or their interaction on storage days 7 and 14, the pH value and antioxidative capacities (only day 14) were comparable (*p* > 0.05) between the pork- and turkey-meat cooked sausage groups. The pork pH values were 5.9 ± 0.10 on day 7 and 5.9 ± 0.01 on day 14 and the turkey meat pH values were 6.0 ± 0.0 on day 7 and 6.0 ± 0.0 on day 14. The mean antioxidative capacity values for pork and products on storage day 14 were 3.7 ± 0.7 µM TEAC/g and those for turkey meat products were 2.3 ± 0.5 µM TEAC/g.

#### 3.2.4. Sensory Analysis of the Cooked Sausages During Storage in Modified Atmosphere Packages

**Figure 4:**
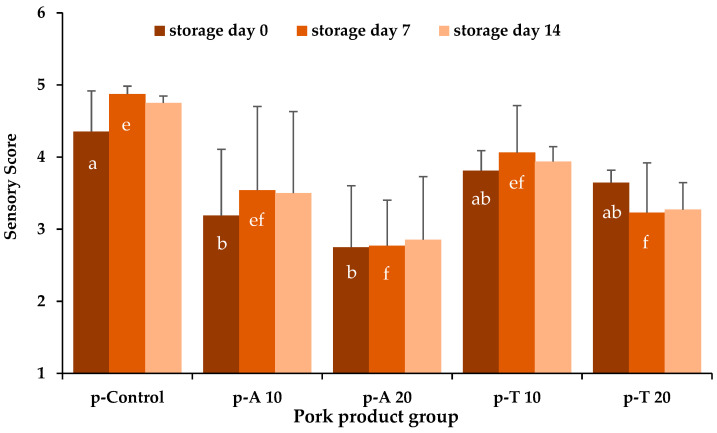

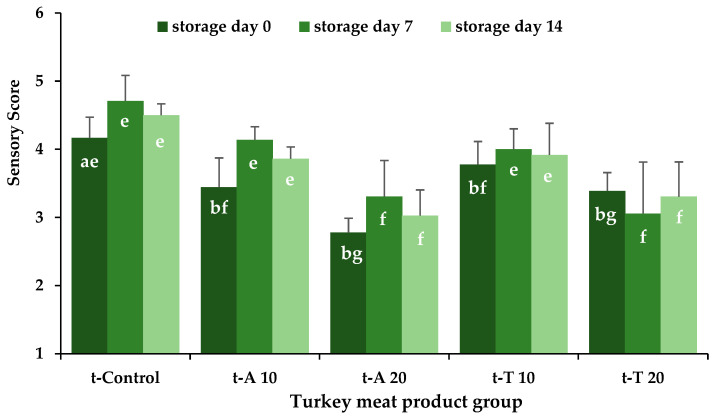
Mean and positive standard deviation values of the sensory analysis of the pork (**above**) and turkey meat (**below**) cooked sausages during storage depending on the meat product group: p/t-control (without insect powder); p/t-A 10/-A 20, consisting of 10%/20% pulverized A. diaperinus larvae; p/t-T 10/-T 20, consisting of 10%/20% pulverized *T. molitor* larvae. The sensory score is given in points from 1 (unacceptable, insufficient quality) to 5 (excellent quality, no deviations). ^ab^ letters mark significant effects considering the insect species (N = 6) and the control (N = 3) (*p* S ≤ 0.05) between the meat product groups; ^efg^ different letters mark significant differences (*p*-value C ≤ 0.05) with regard to the concentrations (0% (N = 3), 10% (N = 6), 20% (N = 6)).

The sensory analysis evaluated the appearance and odor of the cooked pork and turkey meat sausages, resulting in a total sensory score, taking into account the appearance with three times the value compared to the odor. However, no significant differences with regard to the interaction of species and concentration (*p* S*C > 0.05) were found (Figure 4).

With regard to the impact of the insect species on storage day 0, t-control showed significantly (*p* S ≤ 0.05) higher sensory results than all insect sausages. In contrast, on storage day 0, pork control sausages showed only significantly (*p* S ≤ 0.05) higher sensory results compared to the *A. diaperinus* sausages. *T. molitor* pork products did not differ significantly (*p* > 0.05) from *p*-control and the *A. diaperinus* sausages. On days 7 and 14, no significant differences (*p* S > 0.05) between the pork and turkey meat product groups were obtained considering the insect species (Figure 4).

On days 0 and 14, there were no significant differences (*p* C > 0.05) in the sensory results between the pork products groups, considering the insect concentration. On day 7, pork products with 20% insect larvae showed significantly (*p* C ≤ 0.05) lower sensory values compared to p-control. The sensory results of the 10% insect products were comparable (*p* C > 0.05) with those of the other groups. On day 0, all turkey products groups differed significantly (*p* C ≤ 0.05) from each other, with the 0% product scoring the highest, followed by the 10% and the 20% insect products. However, on days 7 and 14 t-control and 10% turkey meat products had comparable (*p* C > 0.05) sensory values whereas the results were significantly (*p* C ≤ 0.05) higher compared to the turkey meat insect products with 20% insect larvae independent of the insect species (Figure 4).

#### 3.2.5. Microbiological Analysis of the Cooked Sausages After Production

With regard to each insect species, its concentration, and their interaction, on all storage days, the microbiological results of the turkey meat and pork cooked sausage product groups did not differ significantly (*p* > 0.05), including both the inoculation experiments as well as the TVC results.

##### Detection of the Total Viable Number of Microorganisms (TVC) and *B. cereus* in the Cooked Sausages During Storage in Modified Atmosphere Packages

The TVC values of the pork products were 1.6 ± 0.3 log_10_ cfu/g on day 0, 1.0 ± 0.2 log_10_ cfu/g on day 7, and 0.7 ± 0.0 log_10_ cfu/g on day 0. The turkey meat products had TVC values of 1.4 ± 0.5 log_10_ cfu/g on day 0, of 1.3 ± 0.01 log_10_ cfu/g on day 7, and of 0.9 ± 0.2 log_10_ cfu/g on day 14.

With regard to the analysis of *B. cereus*, no colonies were ever detected on the pork and turkey meat products.

##### Inoculation Experiments of the Cooked Sausages During Storage in Modified Atmosphere Packages

In the inoculation experiments, during storage up to day 14, *L. monocytogenes* increased by 1.5 log_10_ cfu/g in the cooked pork sausages whereas *C. jejuni* decreased by 1.5 to 2.0 log_10_ cfu/g during storage up to day 14 in the inoculated turkey meat sausage slices. *B. cereus* and *E. coli* decreased by 0.5 log_10_ cfu/g in the cooked turkey meat and pork sausages during modified atmosphere storage.

### 3.3. Analysis of the Cooked Sausages (Impact of the Animal Meat Source)

In this section, some interesting significant differences (*p* M ≤ 0.05) between the meat products considering the two different meat types (pork and turkey meat) and the insect species (*p* M*S ≤ 0.05) are presented.

#### 3.3.1. Nutrient Analysis

After processing, pork products had significantly higher (*p* ≤ 0.05) ash contents than turkey products independent of the insect species (storage day 0 shown in Figure 2).

#### 3.3.2. Fatty Acid Analysis

Independent of the insect species, turkey meat products had significantly higher (*p* M ≤ 0.05) PUFA and lauric acid contents and lower (*p* M ≤ 0.05) MUFA results than the pork products (Table 4).

#### 3.3.3. Physicochemical Analysis

The cohesion and elasticity and pH results of the turkey meat sausages were significantly higher (*p* M ≤ 0.05) and the antioxidative capacities significantly lower (*p* M ≤ 0.05) than those of the pork products.

## 4. Discussion

### 4.1. Native Insect Larvae

At first, it is important to note that a discussion of the nutritional data is difficult as several important factors affect the nutritional values of insects, like feeding, the insect species, and the developmental stage of the insects [8,13,14,15]. In the following sections, we focus on the discussion of the protein, fat, and fatty acid results.

At first, when calculating and discussing the protein content, it should be considered that by using the Kjeldahl technique and a nitrogen-to-protein factor (*kp*) of 6.25, used for lean pork or turkey meat [33], the protein content of insect larvae is often overestimated. The overestimation is caused, on the one hand, by the distinct amino acid profiles of insects compared to lean meat [34], and on the other hand, by the presence of chitin as a part of the exoskeleton. Chitin is a component of non-protein nitrogen (NPN), which is determined during Kjeldahl analysis but must not be included in the protein calculation [35]. For insect larvae, currently, modified *kp* values have been published: for example, by Boulos et al. [35] for *T. molitor* larvae, with a *kp* of 5.33 (±0.10); by Janssen et al. [36], with a *kp* of 4.76 for all whole insect larvae and a *kp* of 5.6 for protein extracts; or by Perez-Santaescolastica et al. [30], with *kp* values of 5.2 and 6.43 for *T molitor* and *A. diaperinus*, respectively. In the present study, by using the nitrogen–protein factors of Perez-Santaescolastica et al. [30], whole *T. molitor* larvae were found to have a protein content of 16.5 ± 1.0% and whole *A. diaperinus* larvae a protein content of 18.6 ± 2.6%. In many publications, the protein results are related to the dry matter content. Therefore, to compare the values, the already published protein results need to be recalculated to the whole larvae protein values, like in the present study considering dry matters of 37% to 39%. Considering this, the protein contents of the examined larvae in the present study are comparable with those of other studies [13,37,38].

The lipid contents of the whole insect larvae in the present study are also comparable with those of other studies [38,39]. The significantly different lipid contents of *T. molitor* and *A. diaperinus* larvae are difficult to explain and should not be overestimated.

Considering the fatty acid percentages, which have not been often published up to now, for example, other studies presented comparable higher ratios of SFAs: UFAs (saturated: unsaturated fatty acids (MUFAs+PUFAs)) of different insect species between 0.43 and 0.79 [40] or between 0.3 and 0.4, especially for *T. molitor* [38], whereas in the present study, the ratios were 0.25 for *T. molitor* larvae and 0.47 for *A. diaperinus* larvae. The present study showed that insects are a good source of UFAs, with high contents of alpha-linolenic acid (C 18:3, omega-3 fatty acid), linoleic acid (C 18:2, omega-6 fatty acid), and oleic acid (C 18:1), which was supported by Syahrulawal et al. [38]. It is known that fish or vegetable oils are a good source of omega-3 fatty acids, and the content of alpha-linolenic acid (C 18:3, omega-3 fatty acid) could be compared to those of krill oil and olive oil and was just slightly lower compared to that of fish oil [41]. Compared to pork [42,43] and turkey meat [43], the examined insect larvae had comparable values with regard to SFA, whereby *T. molitor* larvae tended to have lower SFA values compared to turkey meat or pork. MUFA values in insects were comparable to those in pork [40,42] and notably higher than in turkey meat [43]. The PUFA content was also higher in the insects examined in this study than in pork or turkey meat [40,42]. The higher lauric acid content in *T. molitor* than in *A. diaperinus* larvae is difficult to explain, especially as Mattioli et al. [44] showed higher lauric acid values in *A. diaperinus* than in *T. molitor* larvae. However, the differences might have been again due to the varying endogenic and exogenic factors such as the feed, influencing the nutrient, especially fatty acid, compositions in the two studies [8,13,14,15].

Thus, disorders like brain neuroinflammation or coronary heart disease may be linked to an incorrect, or less beneficial, ratio in the daily diet (predominant ingestion of omega-6 fatty acids) [45]. To enhance PUFAs’ health-promoting effects in products, the *‘Allipids’* project is examining the degree to which unsaturated fatty acids can be stabilized in triple emulsions in order to be able to be integrated into consumer-facing products [46]. As the nutritional makeup of insects is heavily influenced by their diet, further research on the nutrition of insects during their rearing stages could be adapted to achieve an ideal ratio of fatty acids. These can further on be processed into certain products, such as meat products, with a possible nutrient benefit of providing omega-3 fatty acids, which are known to promote health.

The microbiological values (TVC and yeast/molds) of raw insects in the present study agreed with those of other studies [25,44,47]. Accordingly, the microbial load depends on the feed substrates [47] and on the environment. According to the European regulation (EU) 2023/58 for *A. diaperinus* and the regulation (EU) 2022/169 for *T. molitor*, the frozen larvae must not be contaminated with more than 10^5^ cfu/g (TVC) and with more than 10^2^ cfu/g yeast and fungi. The higher microbiological results of the latter in both insect species and the TVC in *A. diaperinus* might have been due to the fact that in insects (larvae), the risk of contamination with feces can only be accomplished through fasting (for 24 h), which is often not a proper way to reach adequate microbial reduction [44,48], in contrast to the slaughter of pigs or turkeys, where the gastrointestinal tract is removed, thereby reducing the risk of microbiological contamination. This possible problem of increased microbiological contamination should be considered if consumers handle insects before preparation/heating or even eat raw insects. This problem is less relevant if (frozen) insect larvae are added to cooked sausages like in the present study, except with regard to heat-resistant bacteria species like *Bacillus* spp. or *Clostridium* spp.

### 4.2. Cooked Sausages (Impact of the Insect Species, Insect Concentration, and Their Interaction)

In the following sections, the results of the cooked sausages, produced with and without insects, determined directly after production (day 0) and during MAP storage (days 7 and 14), will be discussed together to partly prevent duplications.

#### 4.2.1. Nutrient Analysis

As there were no significant differences in the ash, protein, or lipid contents between the manufactured pork and turkey sausages (with or without the addition of insect larvae), it is reasonable to assume that insect larvae contents of 10% and 20% are probably too low to affect a sausage’s nutritional composition. The nutrient values of the whole raw insects, determined in our study, regarding the ash, protein, and lipid contents, were principally comparable to that of pork [43,49] and turkey meat [43,50]. Nevertheless, compared to pork and turkey meat, the larvae examined in this study showed a tendency towards higher values in their lipid contents and a tendency towards lower values in their protein contents. In contrast to the present study, higher protein and lipid concentrations have been seen in meat products with increasing levels of pulverized *T. molitor* larvae [6] and dried, pulverized *Bombyx* (*B.*) *mori* (*Bombyx mori*, Linnaeus, 1758) pupae [51]. Other studies, using defatted and hydrolyzed insect flours [27], as well as insect protein extracts [29], found increased protein concentrations in the finished products as the insect contents increased. However, it should be mentioned that insect protein extracts [29], hydrolysates, or defatted powders [27] from insects, which are frequently used in studies, show higher protein contents than raw, native insect larvae, which might explain the missing significant effect between the insect hybrid and control products in the present study [52]. The extraction of lipids and precipitation of proteins from insect powders, represent important steps in achieving a high and pure protein yield in insect extracts. However, the protein solubility of insect powders depends on the pH value during these extraction steps. Various methods for the processing of insects into powders or extracts have been investigated in order to optimize these extracts, reaching protein contents of more than 90%, which are almost fat- and chitin-free [52,53].

#### 4.2.2. Fatty Acid Analysis

The higher lauric acid content in *T. molitor* larvae (Table 3) might be the reason for the higher lauric acid values in the pork products. Due to the generally higher lauric acid content in turkey meat, which was also shown by Lisitsyn et al. [54], the turkey sausages in our study therefore had higher lauric acid contents than the pork products (Table 4). Accordingly, *T. molitor* larvae had no impact on the lauric acid content in the turkey product. Despite differences in some fatty acids (e.g., palmitic acid, linoleic acid) or SFAs and PUFAs between the insects (Table 3), unfortunately, no significant differences in these parameters, independent of lauric acid, were found in the cooked sausages after insect addition (Table 4). Once more, it is likely that 10% and 20% insect substitutions will not be enough to have significant effects. Other reasons might have been, on the one hand, that the variation in the fatty acid values was partly quite high and, on the other hand, that the main fat source was pork fat.

#### 4.2.3. Physicochemical Analysis

Cooking loss is one important component that influences the determination of the moisture binding capacities of sausages. The present study did not show any significant differences in cooking loss results considering the manufactured meat products, yet 20% turkey–insect hybrid products tended to have a higher cooking loss (*p* C = 0.0529). As high cooking losses can cause financial losses for the producer, this indicates that insect addition is not problematic with regard to this parameter. Scholliers et al. [55] reported that 10% (*Z.*) *morio* hybrid products had a lower cooking loss than meat products without insect contents. Although the tendentially higher cooking loss in the 20% turkey–insect hybrid products in the present study should not be overestimated, it should be generally kept in mind by sausage producers who want to use insects in products that, for example, with the replacement of meat by insects, myofibrillar protein levels are decreased, which can have an impact on the water binding capacities of the products as the co-gelation characteristics of the myofibrillar proteins influence both the meat product’s ability to retain water as well as its related textural qualities [29]. Additionally, chitin, the main component in insects, is a N-acetylated aminopolysaccharide, which is not particularly soluble in water due to its hydrophobic properties [56], and therefore, this may also have a negative impact on the moisture binding. For example, the dried larval form of *T. molitor* consists of approximately 4.6% chitin [56]. The EFSA Panel on Nutrition stated in 2022 [57] that the range of chitin concentration in *A. diaperinus* larvae is 2.6 to 9.1%. One way to improve the meat batter’s moisture binding could be the application of high-pressure processing (HPP) [58], which results in a better protein dissolving and unfolding. In a study, by employing HPP, the texture was enhanced and the cooking loss was decreased [58]. Nevertheless, due to non-significant differences, *T. molitor* and *A. diaperinus* larvae are well suited for the production of cooked sausages in terms of cooking loss.

Han et al. [59] found that sausages containing cricket flour (pulverized, freeze-dried adult crickets) had different texture results compared to sausages without insects, even if only small amounts of cricket flour were used. When utilizing *Z. morio* larvae (superworms) in pork cooked sausages, Scholliers et al. [60] verified this. However, they also discovered that raising the brewing temperature to +80 °C improved the texture while the sausages’ consistency was still inferior to that of the control sausages, containing no insects. As, in the present study, no significant differences between the products in terms of texture were calculated, we were unable to confirm the above mentioned results, probably due to the use of other insect species. An explanation for the quite-high standard deviations evaluating the textural analysis may be deviations in the quality of the raw material (meat and fat). It is known that fat or meat—for example, such as in insects—depend on various factors such as feeding and fattening conditions, and therefore, the texture is subject to minor differences. However, the turkey and pork sausages in this study tended to become softer with increasing insect contents (with the exception of elasticity and cohesion in the 10% lean pork substitute) and showed less cohesion and less elasticity. Choi et al. [6] showed reduced cohesion in frankfurters with 30% *T. molitor* and reduced hardness after the addition of a 5% insect content. Kim et al. [27] described increased hardness values in products containing *T. molitor* larvae and *B. mori* pupae and explained these effects with higher solid contents in the products. If the addition of insects in manufactured sausages is higher (>20%), it might be useful to add additives, like hydrocolloids or psyllium husks, to influence the impact of the insects on the sausage textures [60,61].

In the present study, the insect hybrid products had higher b* (more yellow color) and lower a* values (less redness) in contrast to the control sausages, which might have had an effect on customers’ purchasing decisions. Our sensory analysis support this assumption since, in the insect hybrid products, mainly those containing 20% insect powder, a reduced sensory score was determined by the panel compared to the control products. Various studies agree with these color effects regarding lower L* [6,27,29,51], higher b* [6,27,29], and lower a* [27,51] values in products containing insects. In contrast, Choi et al. [6] described higher a* values in products containing increased numbers of *T. molitor* larvae. Larouche et al. [62] investigated killing methods and their effects on the colors of *H. illucens* larvae but did not subsequently process them in a cooked sausage. It might be useful in further studies to test whether the method of killing *T. molitor* and *A. diaperinus* larvae affects the color of sausages partly produced with these insects.

No significant differences were found in the present study considering the antioxidant capacities of the cooked sausages. The antioxidant capacity is related to the presence of substances like β-carotenes, vitamin A (retinol), C (ascorbic acid) and E (tocopherol), phenols, and other antioxidant-active compounds. Because of their double bonds, UFAs are more easily oxidized [63], probably resulting in increased rancidity in the products, which might reduce the antioxidant capacities. The lower quantities of UFAs in raw *A. diaperinus* compared to raw *T. molitor* (Table 3) had no effect on the contents of the manufactured sausages because the UFA levels in the cooked sausages were comparable (Table 4). That is why a missing significant effect on the antioxidative capacities is plausible. Again, it is possible here that 20% replacement of meat by insects is too little to have a significant effect.

#### 4.2.4. Sensory Analysis

Sausages containing insect powder (independent of the examined insect species *A. diaperinus* and *T. molitor*) received lower sensory scores than the corresponding control meat products. This fact was confirmed by various studies that had produced cooked sausages [64] and frankfurter-style sausages [6,65]. Cruz-Lopez et al. [64] observed a reduced acceptance of cooked sausages with flour from *Sphenarium purpurascens* (grasshopper, *Sphenarium purperascens*, Charpentier, 1842). An increasing content of *A. domesticus* in frankfurters [66] or *T. molitor* [6] reduced acceptability and appearance and resulted in a different coloring compared to the conventional products without insect contents. The larvae of *T. molitor* are described to have a nutty, umami-like flavor. The odor becomes less pleasing as the size of *T. molitor* powder particles increases [67]. Therefore, care should be taken to keep the particle size of max. 1 mm (as small as possible) for pulverized insect larvae before processing these powders in meat products. The higher b* values in insect products (’yellow mealworm’) [6] in combination with decreases in redness (lower a*) and lightness (lower L*) may have contributed to the poorer sensory evaluation and could have had an impact on costumers’ purchase decisions. Upon examining the meat products over the storage period of 14 days, it became evident that storage conditions (modified atmosphere packaging at +6 °C) had minimal to no impact on the sensory attributes for the respective product groups.

#### 4.2.5. Microbiological Analysis

To determine whether it is realizable to add pulverized insect larvae to meat products without an initial pre-treatment, like heating, and the consequences this processing has on technological, physico-chemical, and microbiological parameters were some of the goals of the current study. Although motile bacteria were found on the TVC plates, we could not include these involved agar plates in the analysis as the swarming colonies might have falsified the results. The affected sausages were mainly those containing *A. diaperinus*. As shown in Table 2, *A. diaperinus* larvae had about a 2.0 log_10_ level higher bacterial count than *T. molitor*, which could explain the microbial contamination of the sausages (even after the cooking process, which occurred at a core temperature of +72 °C). Numerous studies that produced insect hybrid meat products did not examine the microbial quality of the end products [6,27,28,55,60]. However, some studies used pre-processing methods like heat treatment [6,27,28,68,69] or high-pressure processing (HPP) [69]. Campbell et al. [69] detected an improved reduction in the TVC during thermal treatment compared to HPP. Our findings and other studies indicate that greater energy input is necessary for pre-processing the insects, such as in the form of autoclaving, blanching, or HPP, to guarantee flawless microbiological quality.

During the storage period in the MAP, the addition of insect larvae had no significant effect on the bacterial growth on the sausage slices previously inoculated with different bacterial species. Since *L. monocytogenes* proliferated in the all sausages during the storage period, it can also be ruled out that the insect component was responsible for the proliferation of *L. monocytogenes*. The fact that *L. monocytogenes* concentrations increased during the storage period while the other inoculated bacteria species (*E. coli*, *B. cereus*, and *C. jejuni*) decreased in concentration was caused by the growth properties of *L. monocytogenes*, which grows at temperatures between −1.5 and +45 °C [70] and at a high fat content in products, like in the emulsion sausages in the present study [71]. In contrast, *B. cereus* [72] and *E. coli* [73] prefer temperatures between +8 and +50 °C. The modified atmosphere in the packages with 30% CO_2_ was mainly responsible for the decreasing bacterial growth in the packages with *E. coli* and *B. cereus* in addition to the temperature-related growth inhibition (primarily of *C. jejuni*) of these bacteria species compared to *L. monocytogenes*.

### 4.3. Cooked Sausages (Impact of the Meat Source (Turkey Meat or Pork))

The higher ash content in pork products compared to that in turkey products is difficult to explain since the ash content in the present study was mainly influenced by the addition of NaCl during the sausage production and as the nutritional values of the meat depended, among other things, on the feed [49,74]. Barbin et al. [50] found an ash content in turkey legs of 1.13 ± 0.01% whereas Ng et al. [49] examined an ash content in pork of 1.03−1.2%, which might present a possible examination, but the results should not be overestimated.

The higher values of PUFAs and lower ones in MUFAs in turkey products were due to the fact that turkey meat has higher PUFA (28%) and lower MUFA (21%) contents compared to pork (PUFAs 11%, MUFAs 46%) [43]. Lisitsyn et al. [54] reports a lower lauric acid content in pork compared to turkey meat, which might be the reason for the higher values in turkey meat sausages (Table 4). Again, the nutrient values and, especially, the fatty acid composition in lean meat can vary depending on the conditions of rearing and the age of the animal, among other factors.

The improved cohesion and elasticity in turkey products when compared to pork sausages may have been related to the significantly higher pH values of the turkey sausages compared to the pork ones. While Choi et al. [6] linked higher pH values to increased cooking losses, Ho et al. [75] showed higher cooking losses with comparable pH values. According to Klettner et al. [76], higher pH values, such as in turkey boiled sausages, can boost the water binding ability and therefore achieve a more elastic texture as presented in the present study.

The lower antioxidant capacity results in the turkey meat sausages might have been due to generally lower levels of antioxidants and/or a higher oxidative metabolism reducing the antioxidant ingredients. Antioxidant concentrations have not been analyzed in turkey meat and pork in the same study as far as we know. Again, UFAs, consisting of double bonds, are more easily oxidized [63], causing higher risks of rancidity in products, probably resulting in lower antioxidant capacities as antioxidants scavenge reactive oxygen species. Turkey-based sausages had significantly higher levels of PUFAs compared to pork cooked products, which could present an explanation for the lower antioxidant capacity of turkey cooked products. Despite the contradictory MUFA results in the present study, the UFA values also explain the effect of saturation on the antioxidative capacities.

## 5. Conclusions

Raw, powdered larvae of *T. molitor* and *A. diaperinus* as substitutes for lean meat in mortadella-like cooked sausages changed the appearance and smell of insect hybrid products, differing significantly from the control sausages. The different appearances were related to the instrumental color analysis results as lower a*, higher b*, and partially lower L* values of the investigated insect products in comparison to the control sausages were found.

The textural properties between the meat product groups were not significantly affected by the insect species and their concentrations (10%, 20%) and their interactions, which is important as consumers expect almost relatable textures for insect-hybrid cooked sausages compared to sausages without insects. However, the data indicate that turkey meat is a better choice for processing or supplementing with insect powder because it causes a more elastic texture and cohesion in the final products.

As high TVC and yeast/fungi values were found in *T. molitor* and *A. diaperinus* before processing, reductions in the microbial load by an insect pre-treatment before processing into meat products might be necessary, especially as cooking sausages at +72 °C is probably insufficient to secure that these products are marketable and suitable for human consumption.

Even if the insect hybrid sausages vary, especially in color and sensory attributes, the *Tenebrionidae* larvae (*T. molitor* and *A. diaperinus*), in particular, have a potential for use during the processing of sausages with regard to the replacement of the meat. The addition can increase the nutritious values of the product. In order to optimize the nutritional value of hybrid meat products, it is necessary to figure out how to assess the nutritional qualities of insect larvae as food through feeding, consistent rearing times, and other protocols. This will guarantee that the nutritional value remains reasonably stable and the product quality is maintained continuously.

## Figures and Tables

**Table 1:** Recipes/groups of the different cooked sausages and the ingredients used depending on the meat type (pork or turkey lean meat) and the added insect larvae powder: control (without insect processing); A 10/A 20 consisted of 10%/20% pulverized *A. diaperinus* larvae and T 10/T 20 consisted of 10%/20% pulverized *T. molitor* larvae.

Groups/Recipes of Cooked Sausages		*Alphitobius diaperinus*		*Tenebrio molitor*	
	Control	A 10	A 20	T 10	T 20
Lean meat: pork shoulder ^1^ or turkey thigh ^1^	50.0	40.0	30.0	40.0	30.0
Pork fat ^1^	29.0	29.0	29.0	29.0	29.0
Water (frozen) ^1^	21.0	21.0	21.0	21.0	21.0
*Alphitobius diaperinus* ^1^	0	10.0	20.0	0	0
*Tenebrio molitor* ^1^	0	0	0	10.0	20.0
Curing salt (NaNO_2_) ^1^	1.9	1.9	1.9	1.9	1.9
Cutter additive, phosphate-based ^1^	0.7	0.7	0.7	0.7	0.7
Spices ^1,2^	0.53	0.53	0.53	0.53	0.53

^1^ Ingredients (lean meat, fat, water, ice, cutter additives, and spices) are listed in %. ^2^ The spices consisted of sugar (0.05%), white pepper (0.3%), macis blossom (0.07%), ginger (0.05%), coriander (0.03%), and nutmeg (0.03%).

**Table 2:** Mean (M) and standard deviation (SD) values of nutritional and microbial results of the larvae of *A. diaperinus* and *T. molitor* (N = 6).

Nutritional Values in % and Microbial Parameters in log_10_ cfu/g	*A. diaperinus* Larvae		*T. molitor* Larvae	
	M	SD	M	SD
Ash content	1.19	0.31	1.25	0.03
Lipid content	6.84 ^b^	0.85	8.61 ^a^	0.70
Protein content ^1^	18.58	2.55	16.74	0.94
Dry matter	27.86	3.13	29.49	1.08
Total water ^2^	72.14	3.13	70.51	1.08
TVC ^3^	6.09	1.89	4.35	1.88
Yeast/Fungi	2.30	1.34	2.38	1.53

^1^ To calculate the protein contents for *A. diaperinus* and *T. molitor*, the N contents after Kjeldahl analysis were multiplicated with 6.43 and 5.2, respectively [30]. ^2^ Total water calculated: 100 − dry matter; ^3^ total viable count (TVC); ^ab^ values with different letters between the insect species were significant (*p* ≤ 0.05). The nutritional values of whole fresh insects were determined.

**Table 3:** Mean (M) and standard deviation (SD) values of different fatty acids (in %) in the larvae of *A. diaperinus* and *T. molitor* (N = 6).

Fatty Acid	Insect Species (Larval Stadium)			
	*Alphitobius diaperinus*		*Tenebrio molitor*	
	M	SD	M	SD
Lauric acid (12:0)	0.05 ^b^	0.00	0.20 ^a^	0.01
Myristic acid (14:0)	0.74 ^b^	0.08	2.91 ^a^	0.14
Palmitic acid (16:0)	22.81 ^b^	0.64	14.33 ^a^	1.65
Stearic acid (18:0)	7.55 ^a^	0.55	2.40 ^b^	0.47
**SFAs (Saturated Fatty Acids)**	32.17 ^a^	1.03	20.19 ^b^	2.01
Palmitoleic acid (16:1)	0.90 ^b^	0.31	1.78 ^a^	0.10
Oleic acid (18:1)	39.30	2.48	41.75	5.44
**MUFAs (Mono-unsaturated Fatty Acids)**	40.66	2.69	43.74	5.48
Linolelaidic acid (18:2, trans-9)	0.08	0.02	<0.005	n.a.
Linoleic acid (18:2, cis-9,12)	25.11 ^b^	3.16	34.57 ^a^	7.20
Alpha-Linolenic acid (18:3, cis-9, 12, 15)	1.11	0.52	1.36	0.38
Eicosapentaenoic acid (20:5, cis-5, 8, 11, 14, 17)	0.24	0.24	<0.005	n.a.
Docosahexaenoic acid (22:6, cis-4, 7, 10, 13, 16, 19)	0.06	0.04	<0.005	n.a.
**PUFAs (Poly-unsaturated Fatty Acids)**	27.16 ^b^	3.44	36.06 ^a^	7.52
**TFA (Total fatty acid amount)**	20.75 ^b^	3.85	25.48 ^a^	2.37

The detection limit was 0.005% of total fatty acid content. n.a. = not applicable; ^ab^ values with different letters between the insect species were significant (*p* ≤ 0.05). The nutritional values of whole fresh insects were determined.

**Table 4:** Mean and standard deviation values (M ± SD) of different fatty acids in the cooked sausages depending on the meat type (pork or turkey meat) and within the meat type upon the formulation of the products.

Fatty Acid Composition (All Values in %)	Groups of Cooked Sausages									
	Pork Cooked Sausages					Turkey Cooked Sausages				
	Control	A 10	A 20	T 10	T 20	Control	A 10	A 20	T 10	T 20
Lauric acid (12:0)	0.07 ± 0.0 ^by^	0.07 ± 0.0 ^by^	0.07 ± 0.0 ^by^	0.08 ± 0.0 ^ay^	0.08 ± 0.0 ^ay^	0.11 ± 0.0 ^x^	0.09 ± 0.0 ^x^	0.09 ± 0.0 ^x^	0.12 ± 0.0 ^x^	0.12 ± 0.0 ^x^
Myristic acid (14:0)	1.22 ± 0.0	1.22 ± 0.1	1.24 ± 0.0	1.31 ± 0.1	1.33 ± 0.1	1.27 ± 0.1	1.29 ± 0.0	1.31 ± 0.0	1.36 ± 0.1	1.38 ± 0.0
Palmitic acid (16:0)	26.46 ± 0.4	26.16 ± 0.5	26.98 ± 0.9	26.99 ± 1.0	26.57 ± 0.9	26.64 ± 0.6	26.78 ± 0.7	26.56 ± 0.6	26.65 ± 0.6	26.74 ± 0.2
Stearic acid (18:0)	14.55 ± 1.1	14.28 ± 0.7	14.49 ± 1.1	14.41 ± 1.0	14.08 ± 0.6	14.03 ± 0.5	13.68 ± 0.4	13.85 ± 0.2	13.43 ± 0.4	13.45 ± 0.4
**SFAs (Saturated Fatty Acids)**	42.60 ± 1.1	42.10 ± 0.8	43.10 ± 1.8	43.10 ± 1.7	42.37 ± 1.3	42.41 ± 1.0	42.21 ± 0.4	42.19 ± 0.7	41.91 ± 0.7	42.04 ± 0.4
Palmitoleic acid (16:1, cis-9)	2.06 ± 0.2	2.01 ± 0.2	2.09 ± 0.2	2.20 ± 0.3	2.11 ± 0.2	2.18 ± 0.1	2.46 ± 0.3	2.44 ± 0.5	2.46 ± 0.1	2.48 ± 0.1
Oleic acid (18:1, cis-9)	43.41 ± 0.3	43.53 ± 2.0	43.68 ± 0.4	42.45 ± 1.4	43.44 ± 0.6	41.84 ± 0.8	41.97 ± 1.5	41.71 ± 0.5	42.44 ± 1.2	42.53 ± 0.7
**MUFAs (Mono-unsaturated Fatty Acids)**	46.50 ± 0.3 ^x^	46.59 ± 2.0 ^x^	46.84 ± 0.2 ^x^	45.68 ± 1.4 ^x^	46.59 ± 0.7 ^x^	45.16 ± 0.8 ^y^	45.60 ± 1.6 ^y^	45.33 ± 0.8 ^y^	46.10 ± 1.2 ^y^	46.18 ± 0.7 ^y^
Linoleic acid (18:2, cis-9,12)	9.13 ± 1.0	9.58 ± 1.9	8.47 ± 1.4	9.53 ± 2.3	9.40 ± 1.4	10.59 ± 1.6	10.37 ± 1.4	10.46 ± 0.5	10.27 ± 1.5	10.05 ± 0.7
Alpha-Linoleic acid (18:3, cis-9, 12, 15)	0.79 ± 0.1	0.84 ± 0.1	0.70 ± 0.2	0.78 ± 0.2	0.76 ± 0.2	0.83 ± 0.1	0.83 ± 0.1	0.83 ± 0.1	0.77 ± 0.2	0.79 ± 0.1
Eicosapentaenoic acid (20:5, cis-5, 8, 11, 14, 17)	0.01 ± 0.0	0.02 ± 0.0	0.03 ± 0.0	0.01 ± 0.0	0.01 ± 0.0	0.02 ± 0.0	0.02 ± 0.0	0.03 ± 0.0	0.01 ± 0.0	0.01 ± 0.0
Docosahexaenoic acid (22:6, cis-4, 7, 10, 13, 16, 19)	0.02 ± 0.0	0.02 ± 0.0	0.02 ± 0.0	0.01 ± 0.0	0.02 ± 0.0	0.01 ± 0.0	0.01 ± 0.0	0.01 ± 0.0	0.01 ± 0.0	0.01 ± 0.0
**PUFAs (Poly-unsaturated Fatty Acids)**	10.85 ± 1.0 ^y^	11.35 ± 2.1 ^y^	10.05 ± 1.6 ^y^	11.21 ± 2.6 ^y^	10.96 ± 1.7 ^y^	12.43 ± 1.7 ^x^	12.19 ± 1.5 ^x^	12.47 ± 0.5 ^x^	11.99 ± 1.7 ^x^	11.78 ± 0.9 ^x^
**TFA (Total fatty acid amount)**	54.22 ± 3.7	55.53 ± 3.1	52.81 ± 3.3	52.74 ± 2.5	53.48 ± 2.2	53.57 ± 2.2	54.27 ± 2.8	52.98 ± 4.9	54.91 ± 1.8	53.68 ± 1.8

Groups: control (without insect processing); A 10/A 20, consisting of 10%/20% pulverized *A. diaperinus* larvae; T 10/T 20, consisting of 10%/20% pulverized *T. molitor* larvae. The TFA (total fatty acid amount) is specified in % of the original substance of the cooked sausage. The named fatty acids and the contents of SFAs (saturated fatty acids), MUFAs (mono-unsaturated fatty acids), and PUFAs (poly-unsaturated fatty acids) are given as percentages of TFA; the detection limit was defined as showing fatty acid methyl esters under 0.005% of total fatty acid content. ^ab^ values with different letters between the insect species (N = 6) and the control (N = 3) were significant (*p* ≤ 0.05). ^xy^ values with different letters between the animal meat sources (N = 6) (pork versus turkey meat) within the same insect species were significant (*p* ≤ 0.05).

**Table 5:** Mean (M) and standard deviation (SD) values of the cooking losses of the cooked sausages depending on the meat type (pork or turkey meat) and within the meat type upon the formulation of the products (N = 3).

Groups of Cooked Sausages	Cooking Losses in %			
	Turkey Cooked Sausages		Pork Cooked Sausages	
	M	SD	M	SD
Control	0.45	0.06	0.47	0.27
A 10	0.43	0.07	0.52	0.24
A 20	0.49	0.07	0.67	0.24
T 10	0.45	0.03	0.43	0.21
T 20	0.59	0.14	0.47	0.13
*p*-value S	0.2211		0.2112	
*p*-value C	0.0529		0.4144	
*p*-value S*C	0.3837		0.6142	

Groups: control (without insect processing); A 10/A 20, consisting of 10%/20% pulverized *A. diaperinus* larvae; T 10/T 20, consisting of 10%/20% pulverized *T. molitor* larvae. *p*-values show the effects of the insect species and control (*p*-value S), the concentrations (*p*-value C), or the interaction between species/control and concentration (*p*-value S*C); *p*-values value below 0.05 were considered significant.

**Table 6:** Mean (M) and standard deviation (SD) values of the lightness (L*), redness (a*), and yellowness (b*) results of the cooked sausages after production (day 0) and during storage (day 7, 14) in modified atmosphere packages (70% N_2_, 30% CO_2_) depending on the meat type (pork or turkey meat) and within the meat type upon the formulation of the products.

Groups of Cooked Sausages	Color Analysis of the Cooked Sausages After Production (Day 0) and on Storage Days 7 and 14																	
	L*						a*						b*					
	Day 0		Day 7		Day 14		Day 0		Day 7		Day 14		Day 0		Day 7		Day 14	
	M	SD	M	SD	M	SD	M	SD	M	SD	M	SD	M	SD	M	SD	M	SD
Pork cooked sausages																		
Control	73.6	0.8	73.8	1.1	74.2	1.2	12.1^ae^	0.7	11.6^ae^	0.4	11.4^ae^	0.7	11.2^bg^	0.3	11.2^bg^	0.3	11.3^bg^	0.1
A 10	71.9	3.0	70.8	4.4	71.4	3.5	10.1^bf^	0.8	9.7^cf^	0.6	9.9^bf^	0.7	12.4^af^	0.4	12.3^af^	0.1	12.7^af^	0.1
A 20	69.7	2.8	68.4	4.3	69.3	4.0	9.3^bf^	0.4	9.2^cf^	0.3	9.4^bf^	0.5	13.4^ae^	0.4	13.6^ae^	0.6	13.6^ae^	0.4
T 10	73.1	1.8	72.6	2.0	73.0	1.7	10.8^bf^	0.6	10.4^bf^	0.5	10.5^bf^	0.8	12.4^af^	0.8	12.4^af^	0.3	12.6^af^	0.3
T 20	71.4	1.8	71.1	1.2	71.6	1.0	10.2^bf^	0.3	9.9^bf^	0.5	10.0^bf^	0.5	13.7^ae^	0.4	13.4^ae^	1.3	13.9^ae^	0.3
*p* S	0.2010		0.0828		0.0735		<0.0001		<0.0001		0.0017		0.0009		0.0079		<0.0001	
*p* C	0.0914		0.1070		0.0905		0.0001		<0.0001		0.0024		<0.0001		<0.0001		<0.0001	
*p* S*C	0.7641		0.7377		0.8080		0.7586		0.8848		0.9647		0.5646		0.6563		0.2565	
Turkey-meat cooked sausages																		
Control	73.0^e^	0.9	72.8	0.9	72.6	1.1	11.4	1.6	11.9^ae^	0.5	120^ae^	1.0	11.7^f^	0.8	11.2	0.5	11.3^ag^	0.3
A 10	73.2^e^	1.2	72.9	0.9	72.9	1.3	10.4	1.0	10.5^bef^	0.5	10.2^bf^	0.7	12.2^f^	0.1	12.3	0.2	12.4^bf^	0.4
A 20	70.5^f^	0.6	70.8	1.5	70.5	0.3	10.1	0.6	10.2^bf^	0.3	10.1^bf^	0.9	13.5^e^	0.2	12.2	0.5	13.7^be^	0.2
T 10	72.6^e^	0.8	72.0	1.1	72.2	2.0	10.6	0.8	10.9^abef^	0.6	10.4^bf^	0.6	12.0^f^	0.8	12.8	1.8	12.1^bf^	0.4
T 20	71.4^f^	0.5	71.3	2.1	71.4	1.3	9.8	0.6	10.6^abf^	1.5	9.4^bf^	0.8	13.0^e^	0.8	12.4	1.5	13.3^be^	0.8
*p* S	0.7610		0.4436		0.5619		0.9460		0.0157		0.0049		0.3405		0.7483		0.0104	
*p* C	0.0025		0.0938		0.0701		0.3874		0.0189		0.0033		0.0027		0.7712		<0.0001	
*p* S*C	0.1424		0.3508		0.3034		0.6721		0.9091		0.3861		0.8113		0.4489		0.8355	

Groups: control (without insect processing); A 10/A 20, consisting of 10%/20% pulverized *A. diaperinus* larvae; T 10/T 20, consisting of 10%/20% pulverized *T. molitor* larvae. *p*-values show the effects of the insect species and control (*p*-value S), the concentrations (*p*-value C), or the interaction between species/control and concentration (*p* S*C); ^abc^ different letters show significant differences (*p* S ≤ 0.05) considering the insect species (N = 6) and control (N = 3); ^efg^ different letters mark significant differences (*p* C ≤ 0.05) with regard to the concentrations: 0% (N = 3), 10% (N = 6), and 20% (N = 6).

## Data Availability

All data have been shown or noticed within this publication.
